# Emergence of superconductivity in Th_3_P_4_-type Sn_3_Se_4_ and Ge_3_S_4_ under high pressure synthesized by diamond anvil cell with boron-doped diamond electrodes

**DOI:** 10.1080/14686996.2026.2705815

**Published:** 2026-07-23

**Authors:** Ryo Matsumoto, Satoshi Nakano, Kensei Terashima, Toru Shinmei, Tetsuo Irifune, Motoharu Imai, Yoshihiko Takano

**Affiliations:** a Research Center for Materials Nanoarchitectonics, National Institute for Materials Science, Tsukuba, Ibaraki, Japan; b Geodynamics Research Center, Premier Institute for Advanced Studies, Ehime University, Matsuyama, Japan; c Research Center for Electronic and Optical Materials, National Institute for Materials Science, Tsukuba, Ibaraki, Japan

**Keywords:** High pressure, superconductivity, diamond

## Abstract

High-pressure techniques provide a powerful approach for exploring superconducting materials by enabling structural modifications that significantly alter electronic and phonon properties. In this study, we investigate the emergence of superconductivity in Sn_3_Se_4_ and Ge_3_S_4_ with the Th_3_P_4_-type cubic structure using high-pressure synthesis and in situ electrical transport measurements in a diamond anvil cell equipped with boron-doped diamond electrodes. Both compounds exhibit superconductivity, with maximum transition temperature *T*_c_ of 7.9 K at 8 GPa for Sn_3_Se_4_ and 10.5 K at 11 GPa for Ge_3_S_4_. The behavior of the *T*_c_ as a function of pressure and previously reported theoretical calculations, including related compounds, suggest a phonon-mediated BCS-type mechanism for observed superconductivity. These results expand the family of Th_3_P_4_-type superconductors and demonstrate the effectiveness of high-pressure techniques for discovering new superconducting phases.

## IMPACT STATEMENT

Sn_3_Se_4_ and Ge_3_S_4_ are synthesized at high pressure, and superconductivity in both compounds has been reported for the first time.

## GRAPHICAL ABSTRACT

**Figure:**
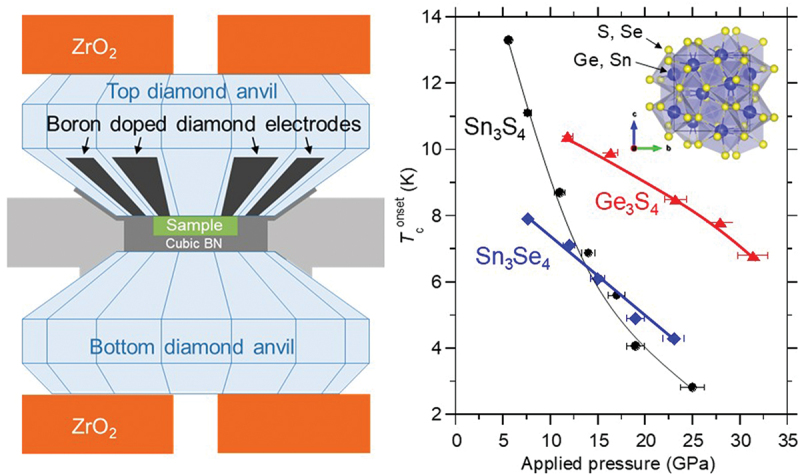

## 1. Introduction

High pressure is a powerful tool in materials science because the crystal structure, which determines the physical properties of a compound, can be manipulated by an external field. During the synthesis process, metastable phases can be obtained by high-pressure annealing, enabling the discovery of novel materials with exotic functionalities [1,2]. In addition, chemical reactions are often accelerated under high pressure due to enhanced atomic diffusion. High pressure also plays a crucial role in tuning the physical properties of synthesized materials through modifications of the electronic and phonon states. One of the most remarkable phenomena induced by pressure is the emergence of superconductivity under compression. For example, hydrogen-based materials synthesized under extremely high pressures above 150 GPa using a diamond anvil cell (DAC) exhibit the highest superconducting transition temperatures (*T*_c_) among all known superconductors [3–5]. In other families of compounds, such as cuprates [6,7], Fe-based materials [8,9], BiS_2_-based materials [10,11], and transition-metal chalcogenides [12], high *T*_c_ values have also been achieved under high pressure. More recently, a new family of high-*T*_c_ superconductors in nickelates, including La_3_Ni_2_O_7_ and La_4_Ni_3_O_10_, has been discovered through high-pressure experiments using the DAC [13,14]. The exploration of superconducting materials utilizing high-pressure techniques has therefore attracted considerable attention as a route to achieving higher functionalities in modern materials science.

A category of materials crystallizing in the Th_3_P_4_-type cubic structure exhibits a variety of functionalities, including high thermoelectric performance [15,16], ultrahigh mechanical properties [17,18], and superconductivity [19]. Among the related compounds, La_3−*x*_Ch_4_ exhibits superconductivity at ambient pressure with *T*_c_ = 8.3 K for La_3−*x*_S_4_ [19,20] and 8.5 K for La_3−*x*_Se_4_ [21]. In addition, Y_3−*x*_S_4_ has recently been reported to be a superconductor with *T*_c_ = 3.6 K and a noncentrosymmetric crystal structure [22]. Under high pressure, the range of substitutable elements in the Th_3_P_4_-type cubic structure expands significantly as metastable phases become stabilized [23]. First-principles calculations have predicted the formation of a metastable Sn_3_S_4_ phase with the Th_3_P_4_-type cubic structure exhibiting superconductivity above 30 GPa [24]. This prediction has been experimentally verified by high-pressure DAC experiments combining structural analysis and electrical transport measurements, which confirmed the formation of the Sn_3_S_4_ phase and the emergence of superconductivity [25]. Following the discovery of superconductivity with *T*_c_ = 12 K in Sn_3_S_4_, the related compound In_3−*x*_S_4_ was investigated to further enhance *T*_c_. Remarkably, In_3−*x*_S_4_ with the Th_3_P_4_-type cubic structure exhibits superconductivity with *T*_c_ = 20 K at 43 GPa [26]. This value represents the highest *T*_c_ reported among superconducting sulfides, except for H_3_S under extremely high pressures above 150 GPa [3]. The pressure-induced superconductivity in the Th_3_P_4_-type cubic family therefore provides an opportunity to explore higher-*T*_c_ materials with relatively simple crystal structures.

In this study, we further extend the compositional range of Th_3_P_4_-type cubic compounds under high pressure using the DAC to investigate the emergence of superconductivity. The candidate materials are Sn_3_Se_4_ and Ge_3_S_4_, which correspond to heavier and lighter analogs of Sn_3_S_4_, respectively. Within the current understanding, the pairing mechanism of superconductivity in this system is considered to be phonon mediated BCS framework [27] and is therefore strongly influenced by the atomic masses of the constituent elements. According to previous reports, Sn_3_Se_4_ has been synthesized by high-pressure heat treatment using laser heating in the DAC, and the emergence of superconductivity based on the BCS mechanism is theoretically predicted [28]. However, its physical properties, including superconductivity, have not been investigated via experiments. Despite the lack of experimental synthesis and theoretical predictions regarding superconductivity in Ge_3_S_4_, the emergence of BCS-type superconductivity is anticipated based on its structural similarity to other Th_3_P_4_-type phases. In this work, we performed high-pressure synthesis and in situ characterization of Th_3_P_4_-type Sn_3_Se_4_ and Ge_3_S_4_ using a custom-designed DAC equipped with boron-doped diamond (BDD) components [29–31]. The formation of these phases was confirmed by synchrotron x-ray diffraction (SXRD) measurements in the DAC during compression. Furthermore, in situ electrical transport measurements were carried out and the emergence of superconductivity in both compounds were observed. The discovery of these superconducting members within this simple crystal structure may stimulate further exploration of functional materials using high-pressure techniques.

## 2. Methods

**Figure 1.**
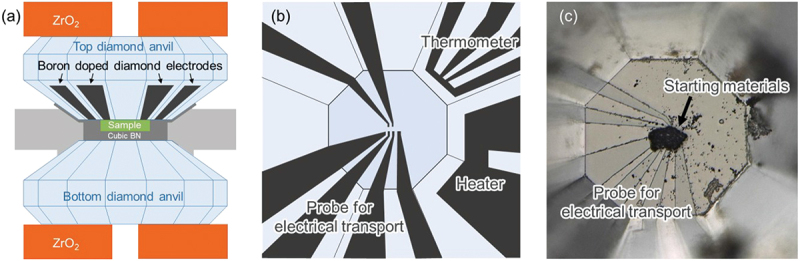
(a) Total configuration of DAC in this study. (b) Schematic image of diamond anvil with BDD electrodes. (c) Typical optical image of the sample mounted on the diamond anvil with BDD probe for the electrical transport measurements.

A custom-designed DAC equipped with BDD components was used for high-pressure synthesis, in situ SXRD analysis, and electrical transport measurements under high pressure. The overall configuration of the DAC is shown in Figure 1(a) [25]. BDD electrodes functioning as a heater and thermometer for sample synthesis, as well as probes for electrical resistance measurements, were fabricated directly on the diamond anvil, as illustrated in Figure 1(b) [25]. The BDD electrode patterns were drawn by using electron-beam lithography in combination with a scanning electron microscope (JSM-5310, JEOL, Japan) equipped with a nanofabrication system (Tokyo Technology, Beam Draw). Epitaxial BDD thin films were grown using a microwave plasma-assisted chemical vapor deposition (MPCVD) technique. During the MPCVD process, the boron concentration in the BDD electrodes was tuned to above ~10^21^ cm^−3^, corresponding to the metallic conduction regime [32,33].

Starting materials of SnSe, SnSe_2_, and GeS were synthesized at ambient pressure using conventional melt and slow-cooling methods from elemental Sn, Se, Ge, and S sealed in evacuated quartz tubes. For the synthesis of Sn_3_Se_4_, a mixture of SnSe and SnSe_2_ with a molar ratio of 2:1 was used, while for Ge_3_S_4_ a mixture of GeS and S with a ratio of 3:1 was prepared. The small cluster of the mixture with a diameter less than 100 μm was placed on the BDD electrodes at the culet of the diamond anvil, as shown in Figure 1(c). After applying the pressure, the mounted sample was electrically contacted to the BDD electrodes. The sample was heated by resistive heating using the BDD heater and thermometer at the desired pressure. The synthesis of Sn_3_Se_4_ was performed at 632 K (18 W) under 31 GPa for the SXRD analysis, and 950 K (26 W) under 23 GPa for the electrical measurement. In the case of Ge_3_S_4_, the synthesis conditions are 915 K (29 W) under 35 GPa for the SXRD analysis, and 967 K (24 W) under 30 GPa for the electrical measurement. The temperature gradually increased from room temperature to the maximum for a few minutes in N_2_ flow conditions, and then rapidly cooled to room temperature.

Single-crystalline and nano-polycrystalline diamond [34] anvils with a culet size of 300 μm were used for the high-pressure generation in the DAC. A rhenium sheet and cubic boron nitride (cBN) served as the sample chamber and pressure-transmitting medium (PTM), respectively. Such a solid PTM with high hardness induces a large pressure distribution inside the sample chamber. The diamond anvils were mounted on ZrO_2_-based ceramic plates with low thermal conductivity in order to minimize thermal diffusion to the cell during sample synthesis. The applied pressure was determined from the fluorescence of ruby powder [35] and from the Raman shift of the diamond anvil tip [36] at room temperature, using an inVia Raman microscope (RENISHAW, UK). The data for pressure calibration are presented in Table S2. Electrical transport measurements were carried out using a four-probe method inside a physical property measurement system (PPMS, Quantum Design, USA) equipped with a 7 T superconducting magnet. A typical probe current was 10 μA.

The crystal structures of the synthesized products were analyzed by SXRD measurements using synchrotron radiation at the AR-NE1A beamline of the Photon Factory (PF) at the High Energy Accelerator Research Organization (KEK). The X-ray beam energy was monochromatized to 30 keV (*λ* = 0.4182 Å). The introduced beam was focused onto the sample in the DAC through a collimator with a diameter of 50 μm. The obtained SXRD patterns were integrated into one-dimensional profiles using the IPAnalyzer software [37]. Background subtraction was performed using QualX2 [38], and lattice parameters were determined using PDIndexer [37]. The LeBail refinement for a phase determination was conducted by Profex software [39]. The bulk modulus of the obtained phases was estimated by fitting the pressure dependence of the lattice volume with a third-order Birch – Murnaghan equation of state using the EOSFIT7 program [40]. Crystal structure visualizations were generated using the VESTA software [41]. The SXRD analysis and the electrical transport measurements were conducted by different DAC with almost all the configurations and synthesis procedures. The number of DAC loadings for the SXRD and electrical measurements was performed once for each compound.

## 3. Results and discussion

**Figure 2.**
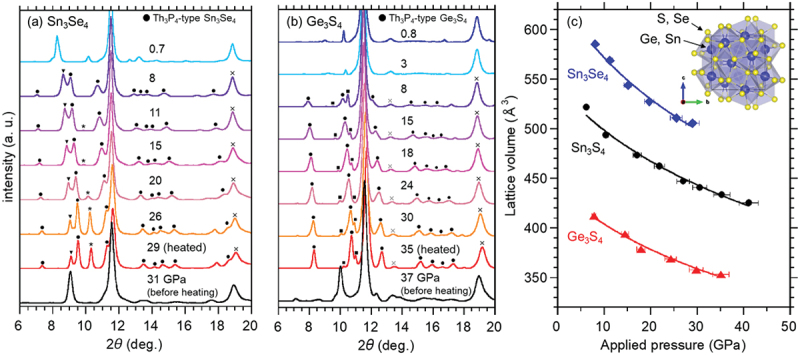
(a) SXRD patterns of the sample after the synthesis at various pressures in the decompression process to obtain Th_3_P_4_-type Sn_3_Se_4_ and (b) Ge_3_S_4_ marked by circle plots. The other phases of SnSe, SnSe_2_, Al_2_O_3_, and cubic BN are indicated by asterisk, triangle, square, and cross markers. (c) Pressure dependence of lattice volume in Sn_3_S_4_, Sn_3_Se_4_, and Ga_3_S_4_ with the fitting curve from the Birch-Murnaghan equation of state [40]. The inserted image is the Th_3_P_4_-type crystal structure, depicted by VESTA [41].

Figure 2(a) shows the SXRD patterns obtained during the high-pressure experiments for Sn_3_Se_4_. After heating the starting material at 31 GPa, several new diffraction peaks appear. The main peaks indicated by the circle markers correspond to the target Sn_3_Se_4_ phase with the Th_3_P_4_-type cubic structure, while the remaining peaks originate from SnSe and SnSe_2_ of unreacted starting materials. Upon decompression, the SnSe phase disappears below 15 GPa. In contrast, the Sn_3_Se_4_ phase remains detectable down to 8 GPa and finally disappears near ambient pressure. The synthesis temperature of 632 K is drastically lower than 1225 K of the previous report. The formation temperature of Sn_3_Se_4_ possibly decreases with increasing pressure by considering the higher synthesis pressure of 31 GPa than 16 GPa in the literature. A similar behavior is observed for Ge_3_S_4_, as shown in Figure 2(b). The main diffraction peaks corresponding to the Th_3_P_4_-type Ge_3_S_4_ phase appear after heating the starting materials at 37 GPa. During decompression, the Ge_3_S_4_ phase disappears below 8 GPa. Diffraction peaks from Al_2_O_3_ and cubic BN used as a pressure marker and a pressure-transmitting medium, respectively, are also observed as components of the DAC assembly. The fitting results of both compounds after the synthesis with a roughly estimated ratio of peak components using LeBail refinement are shown in Figure S1. Because Sn_3_Se_4_ contains two kinds of impurity phases of SnSe and SnSe_2_, the pressure dependence of the peak ratio is also indicated in Figure S2, which is useful to understand the results of physical property measurements.

The lattice volumes of Th_3_P_4_-type Sn_3_Se_4_ and Ge_3_S_4_ were determined by fitting the obtained diffraction patterns and are plotted as a function of pressure in Figure 2(c), together with previously reported data for Sn_3_S_4_. The pressure dependence of the lattice volume was fitted using the Birch – Murnaghan equation of state, assuming the first pressure derivative of the bulk modulus *B*_0_′ = 4.0. From the fitting results, the bulk modulus *B*_0_ and the lattice volume *V*_0_ at 0 GPa were estimated to be 99 GPa and 543 Å^3^ for Sn_3_S_4_, 76 GPa and 636 Å^3^ for Sn_3_Se_4_, and 97 GPa and 440 Å^3^ for Ge_3_S_4_, respectively. Here, the *B*_0_ is sensitive to the change in *B*_0_’ due to the lack of a higher-pressure region, as shown in Figure S5. As expected from the elemental substitution, the lattice volume increases in Sn_3_Se_4_ and decreases in Ge_3_S_4_ compared with that of Sn_3_S_4_. The smaller bulk modulus of Sn_3_Se_4_ indicates relatively higher compressibility compared with Sn_3_S_4_ and Ge_3_S_4_, reflecting the larger ionic radius of Se and the resulting weaker bonding in the crystal lattice.

**Figure 3.**
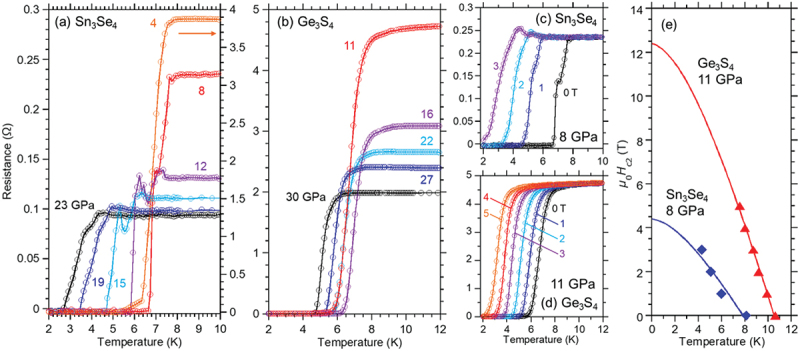
(a) Temperature dependence of resistance in Th_3_P_4_-type Sn_3_Se_4_ and (b) Ge_3_S_4_ under various pressures during the decompression process. (c) The superconducting transition under magnetic field in Sn_3_Se_4_ at 8 GPa and (d) Ge_3_S_4_ at 11 GPa. (e) Upper critical field as a function of temperature with the WHH fitting in Sn_3_Se_4_ at 8 GPa and Ge_3_S_4_ at 11 GPa.

Figure 3(a) shows the temperature dependence of the electrical resistance of Th_3_P_4_-type Sn_3_Se_4_ measured under various pressures during the decompression process. The data for the whole range are presented in Figure S6. At the highest pressure of 23 GPa after the high-pressure synthesis of Sn_3_Se_4_, the resistance sharply drops to zero from 4.3 K, indicating the onset of superconductivity. As seen in Figure S3, we define the three kinds of *T*_c_, which is *T*_c_^onset^, *T*_c_^middle^, and *T*_c_^zero^. In the main discussion, the *T*_c_^onset^ is used because it reflects the maximum *T*_c_ within the large pressure distribution of the sample. The evaluation using another *T*_c_^middle^ and *T*_c_^zero^ is described in Figure S4. The *T*_c_^onset^ of Sn_3_Se_4_ increases monotonically with decreasing pressure and reaches 7.9 K at 8 GPa. The resistance abruptly increases below 8 GPa, which is likely associated with partial decomposition of the phase, as suggested by the SXRD observations. Possible superconducting contributions from the byproducts SnSe and SnSe_2_ must also be considered, since these phases are detected in the SXRD analysis. However, previous studies report that the high-pressure cubic phase of SnSe exhibits a maximum *T*_c_ of 4 K at around 50 GPa [42] and shows only weak pressure dependence. Also, the Sn_3_Se_4_ phase still survives below 10 GPa, although the SnSe phase disappears as seen in Figure S2. Similarly, SnSe_2_ exhibits a maximum *T*_c_ of 6 K near 30 GPa with relatively poor pressure dependence [43]. These behaviors are clearly different from the pressure dependence observed in Figure 3(a). Therefore, the superconductivity observed here is attributed to the synthesized Sn_3_Se_4_ phase. Figure 3(b) shows the temperature dependence of resistance for Th_3_P_4_-type Ge_3_S_4_ measured during decompression. Similar to the case of Sn_3_Se_4_, superconductivity appears at 6.8 K at the highest pressure of 30 GPa following the high-pressure synthesis. The *T*_c_^onset^ increases with decreasing pressure and reaches a maximum of 10.4 K at 11 GPa. One possible candidate for superconductivity is GeS, which was used as a starting material in the synthesis of Ge_3_S_4_. However, GeS becomes superconducting only above 50 GPa, and its *T*_c_ enhances with increasing pressure [44]. The pressure dependence observed in the present experiments is therefore inconsistent with that of GeS, indicating that the superconductivity shown in Figure 3(b) originates from the synthesized Ge_3_S_4_ phase. Although we confirm the superconducting signal solely from the electrical transport measurement without monitoring the Meissner effect in magnetic measurements, the bulk superconductivity possibly appears in both compounds because of the observation of zero resistivity, which is one of the indicators, as discussed in nickelate cases [45]. The magnetization measurement under high pressure is expected as a future investigation using the SQUID magnetometer or the quantum sensor based on the nitrogen-vacancy center in diamond.

The superconducting transition in both compounds is gradually suppressed by applying an external magnetic field, as shown in Figure 3(c,d), providing further evidence that the observed drop in resistance is due to superconductivity. The upper critical field *μ*_0_*H*_c2_(0) was estimated from the temperature dependence of *μ*_0_*H*_c2_ using the Werthamer – Helfand – Hohenberg (WHH) model [46,47]. The *T*_c_^onset^ is used for the fitting. The WHH fitting yields *μ*_0_*H*_c2_(0) values of 4.4 T for Sn_3_Se_4_ at 8 GPa and 12.4 T for Ge_3_S_4_ at 11 GPa, both of which are within the weak-coupling Pauli limit of 1.84*T*_c_. This behavior is consistent with other superconducting compounds with the Th_3_P_4_-type structure under high pressure, such as Sn_3_S_4_ and In_3−*x*_S_4_. In contrast, the isostructural compound La_3−*x*_S_4_ at ambient pressure exhibits a *μ*_0_*H*_c2_(0) value exceeding the Pauli limit [19]. As future research, a direct observation of the *μ*_0_*H*_c2_(0) using the higher magnetic fields without the extrapolation. The coherence length at zero temperature *ξ*(0) is estimated to be 8.6 nm for Sn_3_Se_4_ and 5.2 nm for Ge_3_S_4_ using the Ginzburg – Landau relation *μ*_0_*H*_c2_(0) = Φ_0_ / 2π*ξ*(0) [2], where Φ_0_ is the magnetic flux quantum. All the data for the temperature dependence of *μ*_0_*H*_c2_ and *μ*_0_*H*_c2_(0) are presented in Figure S7 and Table S1, respectively.

**Figure 4.**
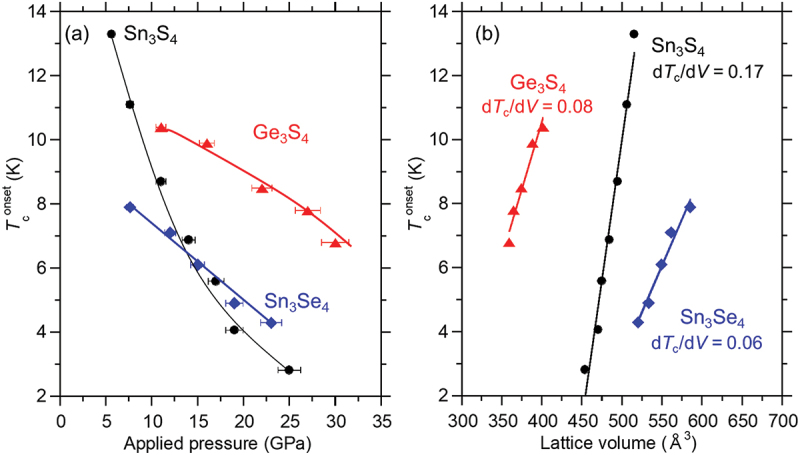
(a) Pressure dependence and (b) lattice volume dependence of *T*_c_ in Th_3_P_4_-type Sn_3_Se_4_ and Ge_3_S_4_ with that of Sn_3_S_4_ as a comparison.

The pressure dependence of *T*_c_ in Th_3_P_4_-type Sn_3_Se_4_ and Ge_3_S_4_ is plotted in Figure 4(a) together with that of Sn_3_S_4_ for comparison. The value of *T*_c_s in these compounds increases monotonically with decreasing pressure. Such pressure dependence of *T*_c_ is commonly observed in phonon-mediated BCS-type superconductors, where lattice expansion enhances the density of states (DOS) at the Fermi energy (*E*_F_) due to a modification of the electronic bandwidth. Figure 4(b) shows the *T*_c_ as a function of the lattice volume *V*. The compounds with a higher *T*_c_ exhibit a steeper d*T*_c_/d*V*, suggesting that a sharp DOS peak exists at the *E*_F_ and contributes to the magnitude of *T*_c_. The maximum *T*_c_ of Sn_3_Se_4_ is lower than that of Sn_3_S_4_, which is consistent with expectations based on the conventional BCS framework. In another cubic phase of SnSn and SnSe, the *T*_c_ in SnSe is obviously lower than that of SnS because of lower Debye temperature from heavy atomic mass. In contrast, Ge_3_S_4_ also exhibits a lower *T*_c_ than Sn_3_S_4_ despite its smaller lattice volume. This deviation from a simple lattice-volume scaling suggests that factors beyond the mass effect in the conventional BCS picture may influence superconductivity in this family. Previous studies reported that Th_3_P_4_-type In_3−*x*_S_4_ exhibits a remarkably high *T*_c_ of 20 K under 43 GPa [26]. The first principles calculations indicate that the superconducting Sn_3_S_4_, Sn_3_Se_4_, and In_3_S_4_ with relatively high *T*_c_ exhibit a comparable contribution of cation and anion bands for *E*_F_ in the DOS. However, almost all the DOS at the *E*_F_ is from only anion in Ge_3_Se_4_ with a low *T*_c_ below 5 K. Considering the present results together with those of related compounds, the use of light element for anion site and the use of relatively heavy element for cation site to realize the comparable contribution of cation and anion bands at *E*_F_ of the DOS are important to increase the *T*_c_ in Th_3_P_4_-type superconductor. The candidate to satisfy these insights are Pb_3_S_4_, which has never been synthesized. The discovery of superconductivity in Sn_3_Se_4_ and Ge_3_S_4_ further expands the Th_3_P_4_-type superconducting family and provides a promising platform for exploring higher-*T*_c_ materials within this simple crystal structure. As a future attempt, the direct observations of the superconducting character of Sn_3_Se_4_ and Ge_3_S_4_ using the theoretical calculation and experimental isotope effect are expected to clear the mechanism for superconductivity.

## 4. Conclusion

In this study, we report the discovery of superconductivity in Sn_3_Se_4_ and Ge_3_S_4_ as new members of the Th_3_P_4_-type cubic family. The samples were synthesized using the DAC equipped with BDD electrodes that function as a heater, thermometer, and electrical probes. In situ SXRD measurements after high-pressure synthesis confirm the formation of Sn_3_Se_4_ and Ge_3_S_4_ as the main phases, with minor amounts of unreacted starting materials. In situ electrical transport measurements reveal clear superconducting transitions with zero resistance in both compounds. The observed *T*_c_ values and their pressure dependences are distinctly different from those reported for possible impurity phases. Based on this comparison, we conclude that the observed superconductivity originates from Sn_3_Se_4_ and Ge_3_S_4_, establishing them as new superconducting members of the Th_3_P_4_-type cubic family. These findings expand the Th_3_P_4_-type superconducting family and highlight the effectiveness of high-pressure synthesis for discovering new superconducting materials.

## Supplementary Material

Supplemental Material
