# Supplementary material for: Emergence of superconductivity in Th3P4-type Sn3Se4 and Ge3S4 under high pressure synthesized by diamond anvil cell with boron-doped diamond electrodes
Source: Sci Technol Adv Mater. 2026 Jul 23;27(1):2705815. doi: 10.1080/14686996.2026.2705815 (PMC13439773; doi:10.1080/14686996.2026.2705815)
Supplement: Supplementary file 1 [file TSTA_A_2705815_SM3754.pdf]

Supplemental Information for

Emergence of superconductivity in Th_3P_4-type Sn_3Se_4 and Ge_3S_4 under high pressure synthesized by diamond anvil cell with boron-doped diamond electrodes

**Ryo Matsumoto[1], Satoshi Nakano[1], Kensei Terashima[1], Toru Shinmei[3], Tetsuo Irifune[3], Motoharu Imai[2], Yoshihiko Takano[1]*

**Corresponding author; Email: MATSUMOTO.Ryo@nims.go.jp*

[1]International Center for Materials Nanoarchitectonics (MANA), National Institute for Materials Science, Tsukuba, Ibaraki 305-0047, Japan

[2]Research Center for Electronic and Optical Materials National Institute for Materials Science, Tsukuba, Ibaraki 305-0047, Japan

[3]Geodynamics Research Center, Premier Institute for Advanced Studies, Ehime University, Matsuyama, Ehime 790-8577, Japan

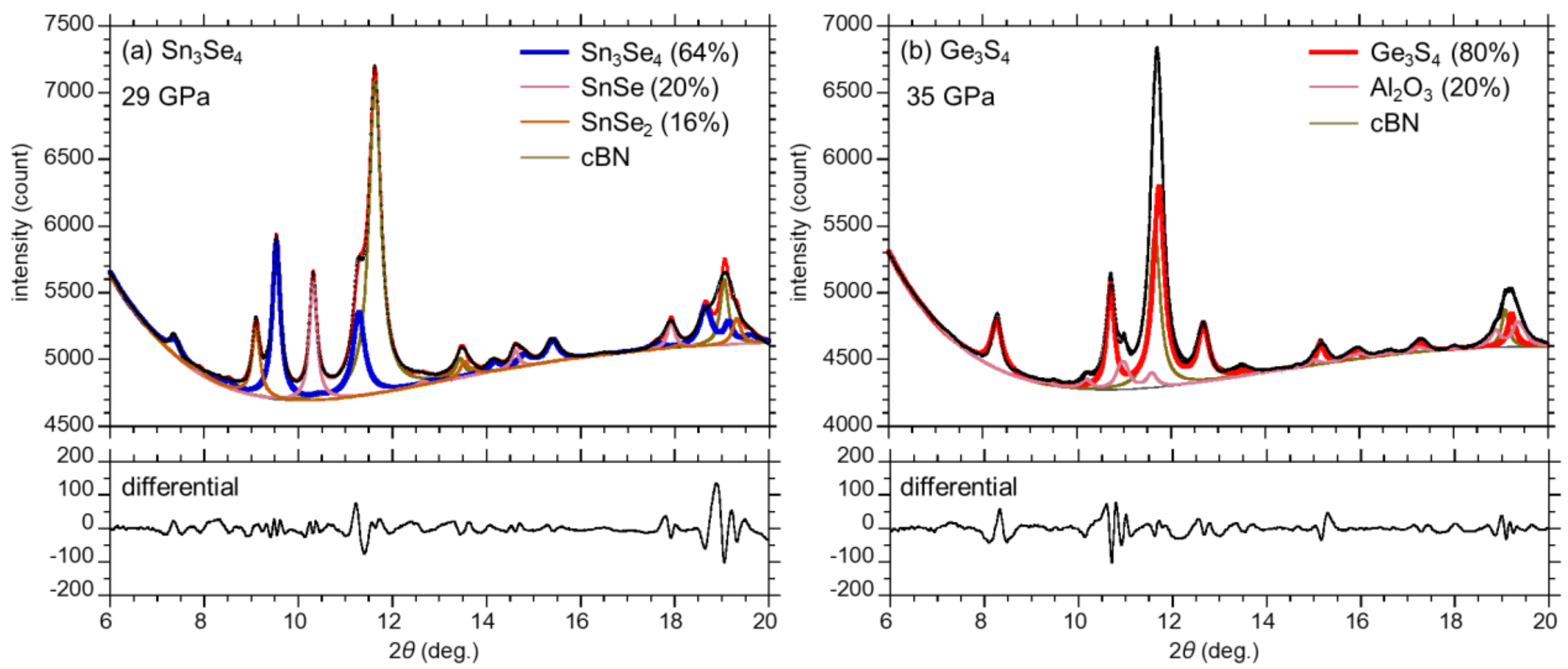

FIG. S1. The typical fitting results of the LeBail refinement for (a) Sn_3Se_4 and (b) Ge_3S_4 after the high-pressure synthesis.

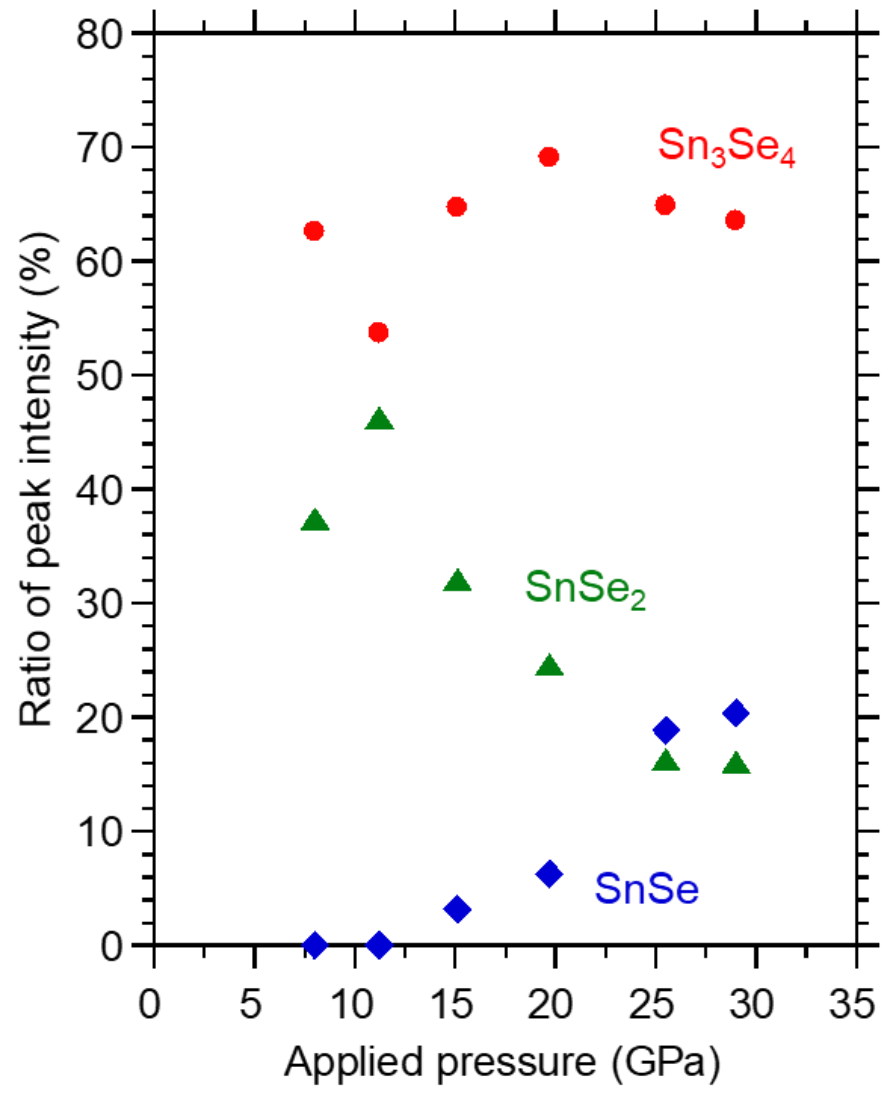

FIG. S2. Pressure dependence of the ratio of peak intensity of each phase in the synthesis of Sn_3Se_4.

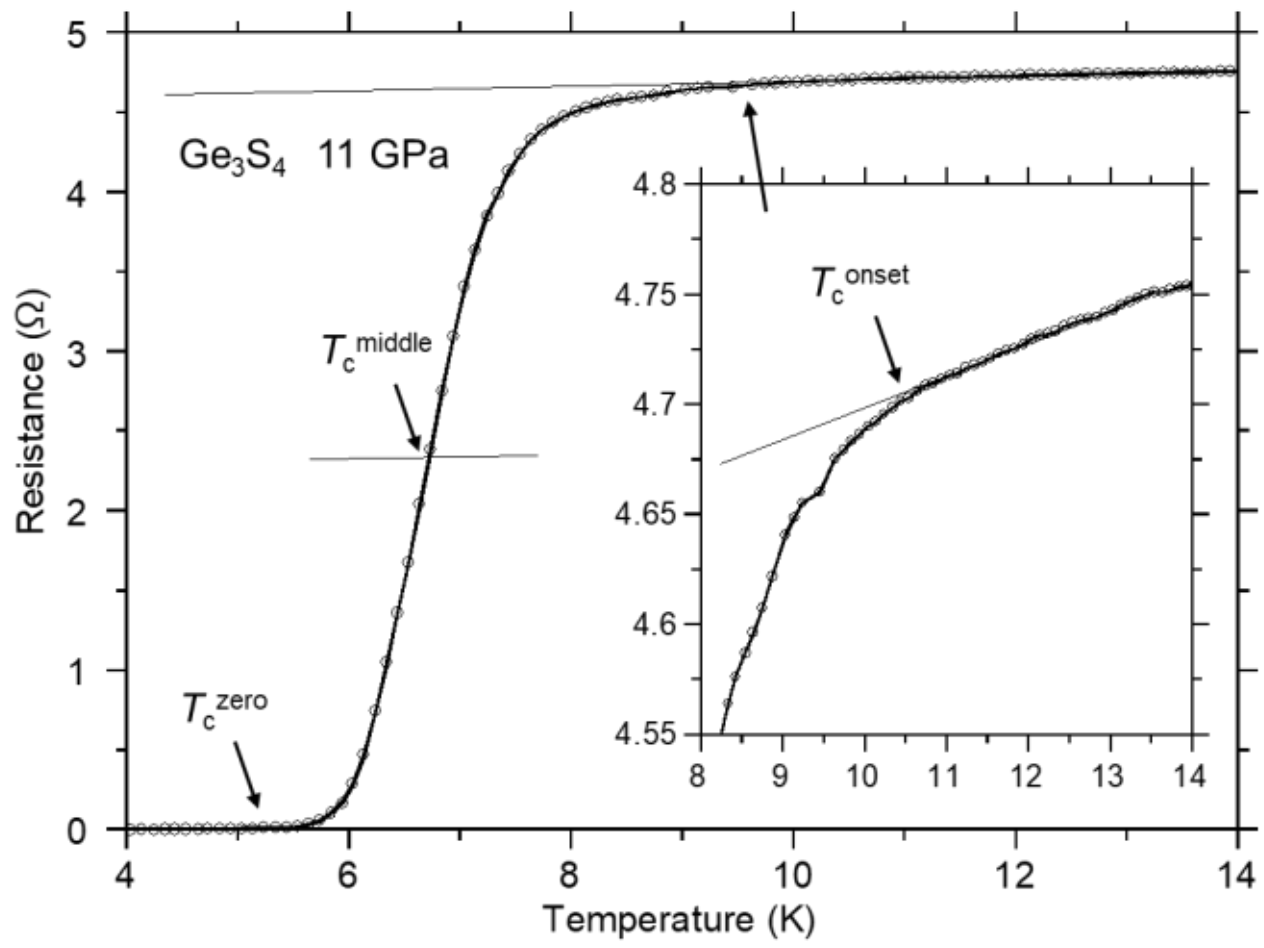

FIG. S3. The determination method for the T_c^{onset}, T_c^{middle}, and T_c^{zero} in this study.

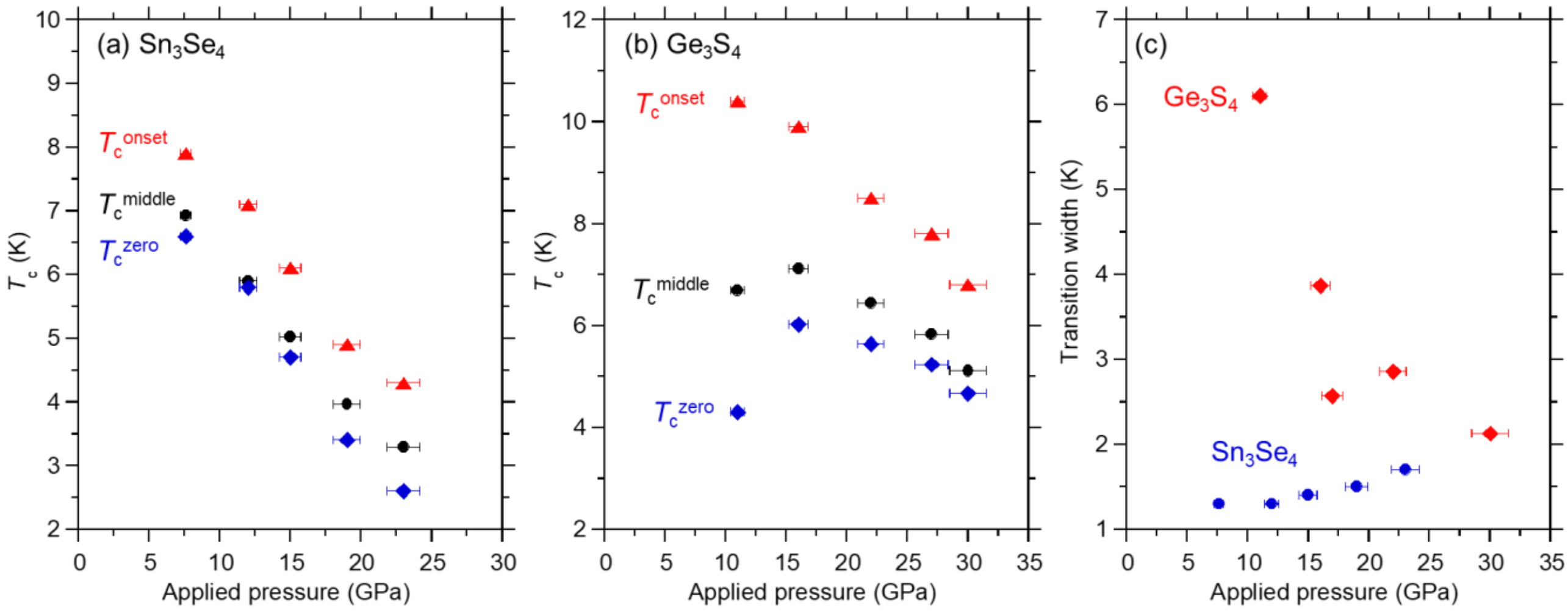

FIG. S4. The pressure dependence of T_c with different criteria for (a) Sn_3Se_4, (b) Ge_3S_4, and (c) transition width (T_c^{onset}-T_c^{zero}).

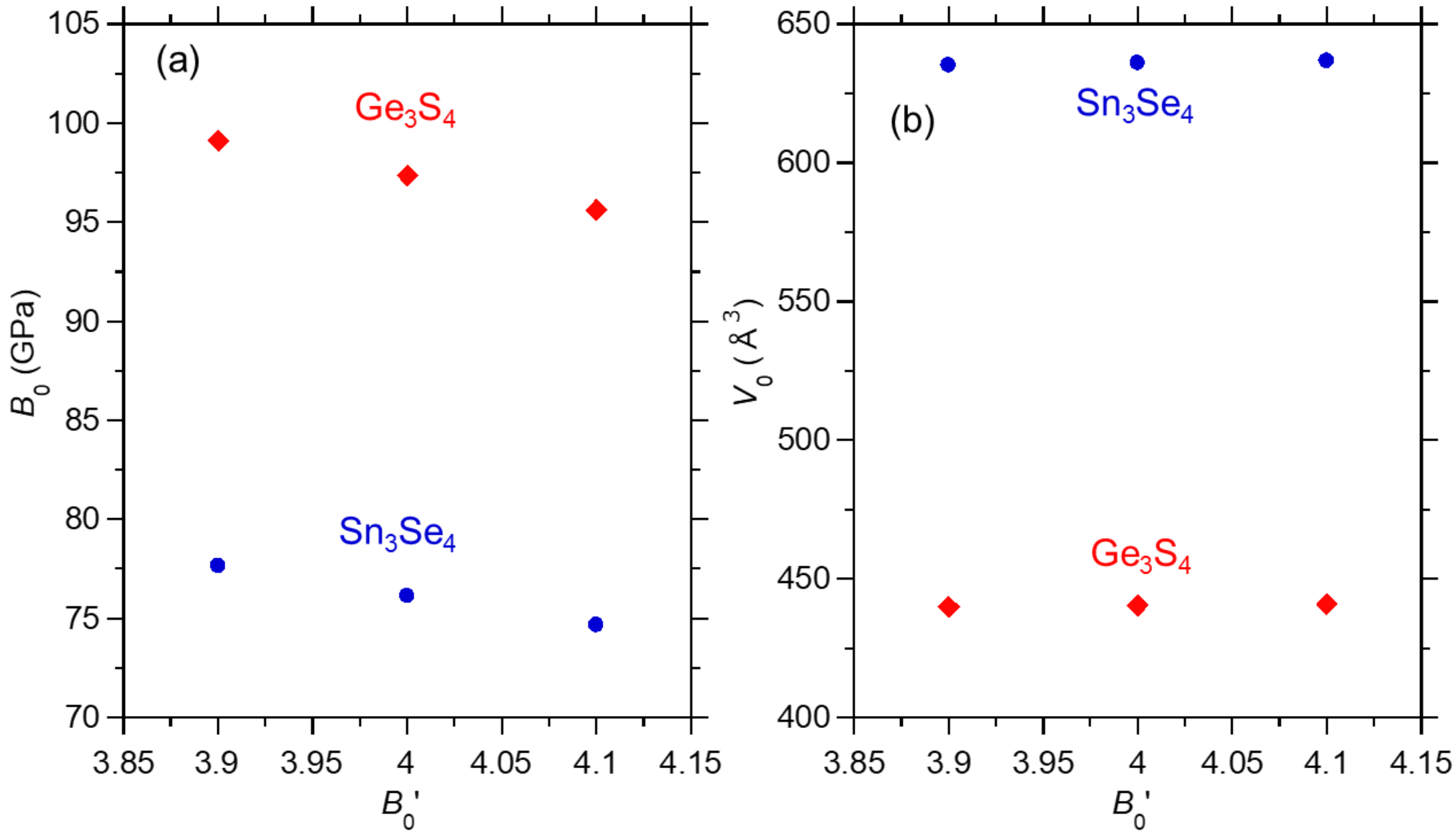

FIG. S5. B_0' dependence of (a) B_0 and (b) V_0 among the fitting parameters of a third-order Birch–Murnaghan equation of state for Sn_3Se_4 and Ge_3S_4.

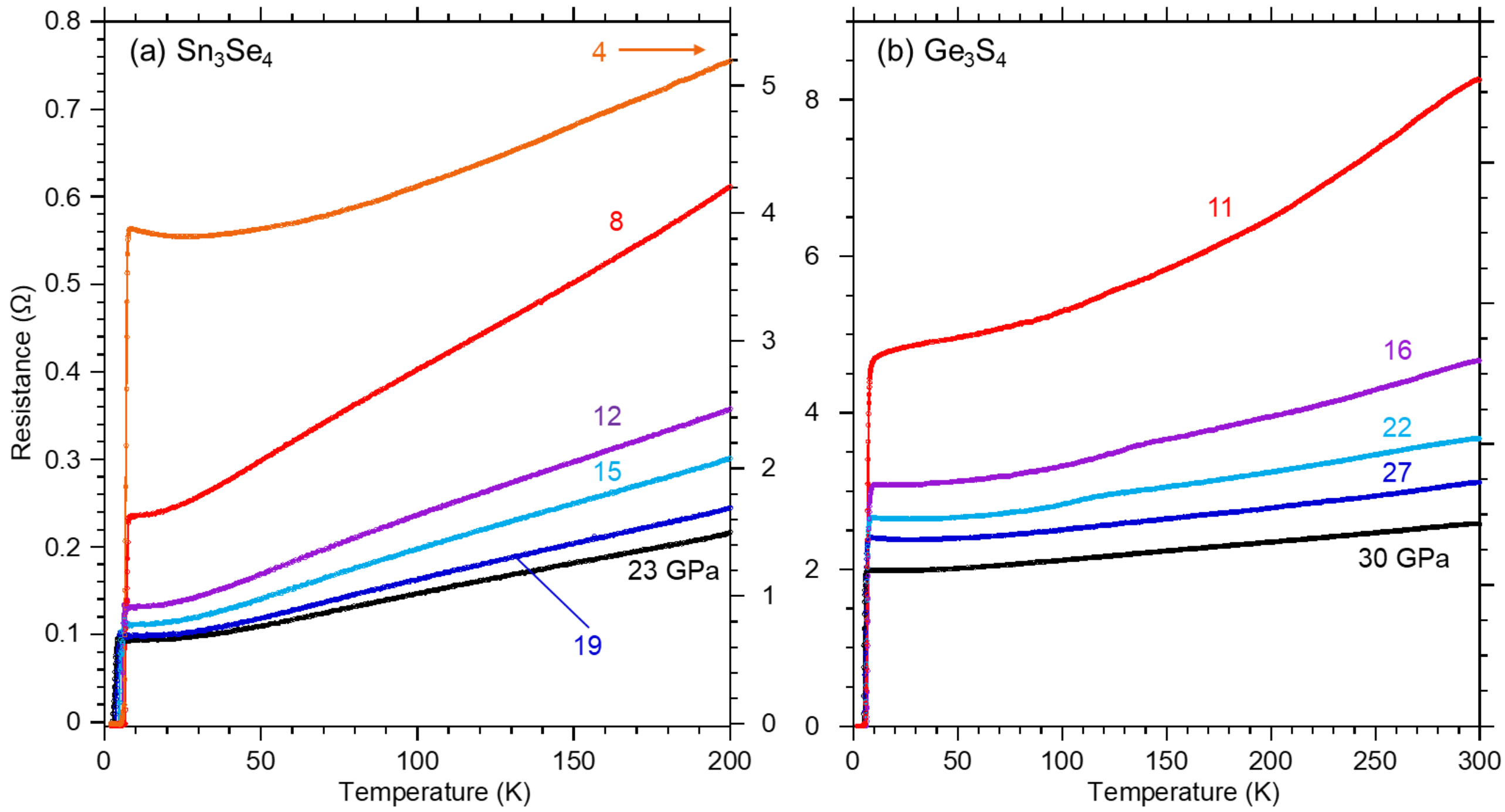

FIG. S6. Whole region of the temperature dependence of electrical resistance under high pressure for (a) Sn_3Se_4 and (b) Ge_3S_4.

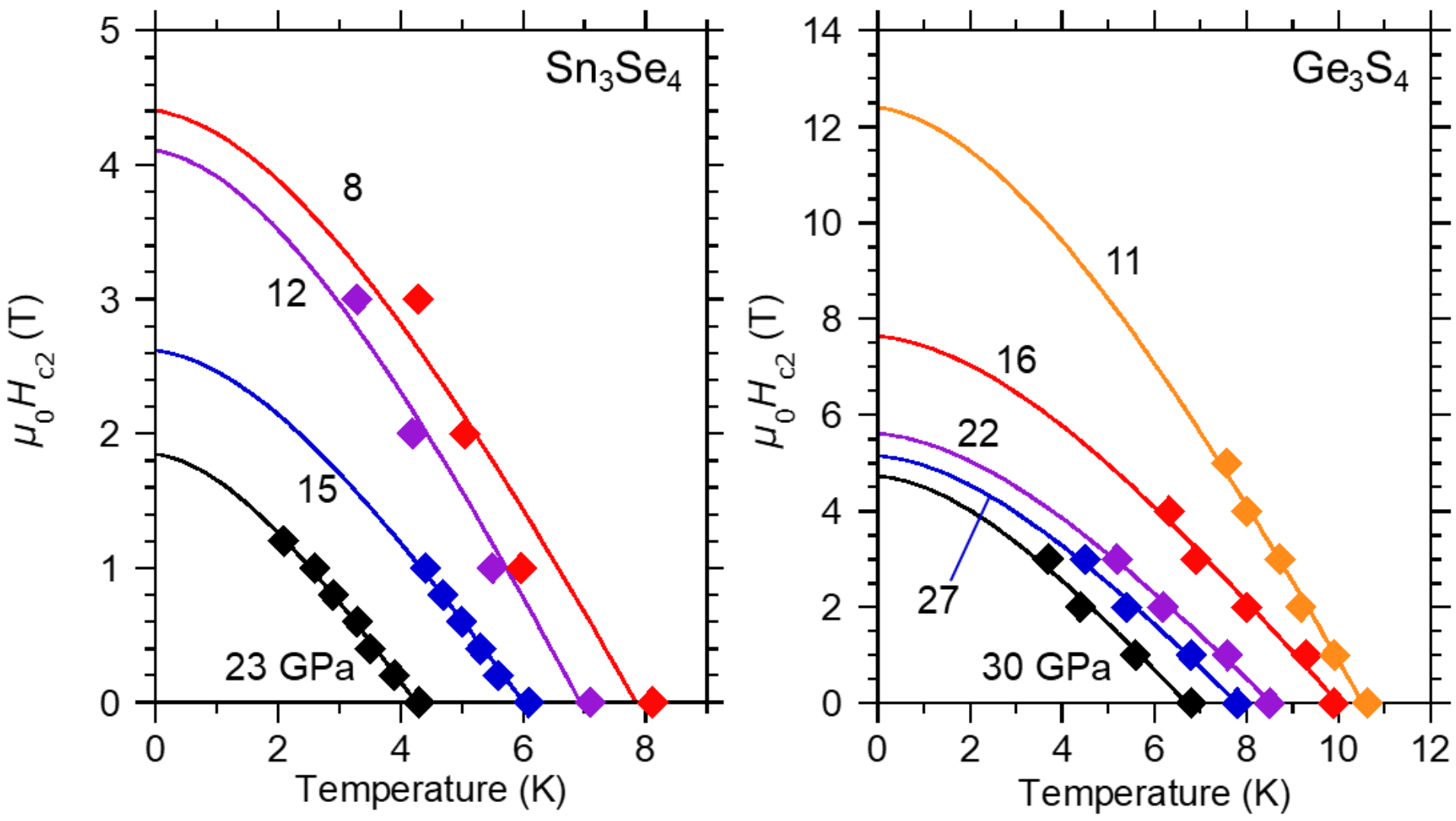

FIG. S6. The temperature dependence of $\mu_0 H_{c2}$ under various pressures of Sn_3Se_4 and Ge_3S_4.

Table S1. List of T_c and $\mu_0 H_{c2}(0)$ under each pressure

Sn_3Se_4			Ge_3S_4		
Pressure (GPa)	T_c^{onset} (K)	$\mu_0 H_{c2}(0)$ (T)	Pressure (GPa)	T_c^{onset} (K)	$\mu_0 H_{c2}(0)$ (T)
23	4.3	1.8	30	6.8	4.7
19	4.9	-	27	7.8	5.1
15	6.1	2.6	22	8.5	5.6
12	7.1	4.1	16	9.9	7.6
8	7.9	4.4	11	10.4	12.4

Table S2. Pressure calibration data

Sn_3Se_4			Ge_3S_4		
Stroke (μm)	Peak position of ruby (nm)	Pressure (GPa)	Stroke (μm)	Raman peak of diamond (nm)	Pressure (GPa)
0	702.5	23	0	1399	30
−40	701.25	19	−40	1394	27
−60	699.7	15	−50	1382	22
−70	698.6	12	−60	1369	16
−80	697.1	8	−70	1359	11
−90	695.9	4			
